# Cupping Therapy May be Harmful for Eczema: A PubMed Search

**DOI:** 10.1155/2013/605829

**Published:** 2013-10-27

**Authors:** Kam Lun E. Hon, David Chi Kong Luk, Kin Fon Leong, Alexander K. C. Leung

**Affiliations:** ^1^Department of Paediatrics, The Chinese University of Hong Kong, 6/F Clinical Sciences Building, Prince of Wales Hospital, Shatin, Hong Kong; ^2^Department of Paediatrics & Adolescent Medicine, United Christian Hospital, Hong Kong; ^3^Institut Pediatrik, Hospital Kuala Lumpur, 50586 Jalan Pahang, Kuala Lumpur, Malaysia; ^4^Department of Pediatrics, The University of Calgary, Alberta Children's Hospital, Calgary, AB, Canada

## Abstract

Eczema is a common childhood atopic condition and treatment is with emollients, topical corticosteroids, and avoidance of possible triggers. *S. aureus* colonization is a common complication. As there is no immediate cure, many parents seek alternative therapies that claim unproven therapeutic efficacy. We report a girl with long history of treatment noncompliance. After practicing a long period of dietary avoidance and supplementation, the grandparents took her to an alternative medicine practitioner. Following cupping therapy and acupuncture, the child developed blistering and oozing over her back the next day, which rapidly evolved to two large irregular-edge deep ulcers. She was treated with intravenous antibiotics and received multidisciplinary supportive intervention. Using search words of  “cupping,” “eczema,” and “atopic dermatitis,” only two reports were found on PubMed. Therapeutic efficacy was claimed but not scientifically documented in these reports. Childhood eczema is an eminently treatable atopic disease. Extreme alternative therapy seems not to be efficacious and may even be associated with serious undesirable sequelae. Physicians should be aware of various alternative treatment modalities and be prepared to offer evidence-based advice to the patients with eczema and their families.

## 1. Introduction

Eczema is a common childhood atopic condition and treatment is with emollients, topical corticosteroids, and avoidance of possible triggers [1]. *Staphylococcus aureus* (*S. aureus*) colonization is a common complication [2, 3]. As there is no immediate cure, many parents seek alternative therapies that claim unproven therapeutic efficacy [4–6]. We report a girl with eczema and long history of treatment noncompliance, who developed two deep ulcers following cupping therapy.

## 2. Case

**Figure 1:**
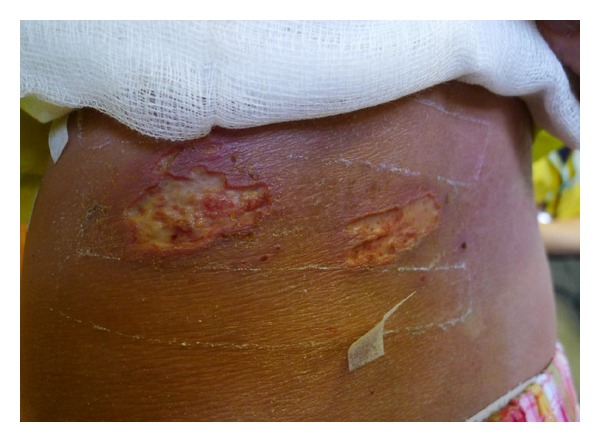
Two deep ulcers with irregular edges over the left flank following cupping and acupuncture in a girl with eczema. Note the lichenified dry skin surrounding the ulcers as a result of long-standing suboptimal treatment and noncompliance.

An 11-year-old girl with complicated social background was cared by her mother and grandparents. She had very high serum IgE of 12455 kIU/L (normal <87 kIU/L). Routine skin prick test showed moderate sensitization to *D. pteronyssinus*, *D. Farinae,* and mixed shellfish but no other food. She also had history of allergic rhinitis and asthma but no history of food allergy. Treatment included emollient, topical corticosteroid, tacrolimus, and antibiotics. Because of difficult-to-treat eczema, *S. aureus* colonization, and noncompliance issues, the grandparents had tried alternative therapies, dietary avoidance, and supplementation, with significant weight loss. She was taken to an alternative medicine practitioner and cupping therapy was tried. Following cupping therapy and acupuncture, the child developed blistering and oozing on her left flank region the next day, which rapidly evolved to two large irregular-edge pusy deep ulcers (Figure 1). The child was admitted to the pediatric ward of a hospital and treated with antibiotic and wound care. Opportunistically, multidisciplinary supportive interventions including plastic surgery, psychology, and dietary therapies were initiated.

## 3. Discussion

**Figure 2:**
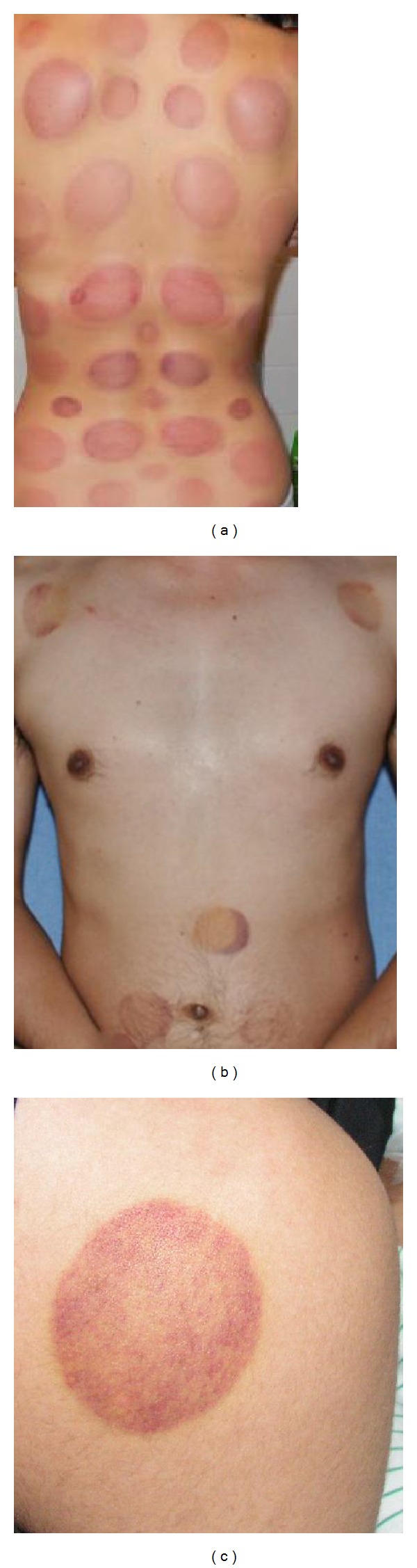
Multiple cupping marks on the back of a lady (a), front of a young man (b), and typical cupping mark on the back of the right shoulder of a boy (c) who sought cupping therapy after an arm sprain.

Cupping therapy is an ancient Chinese form of alternative medicine in which a local suction is created on the skin [7]. A partial vacuum is created in cups placed on the skin either by means of heat or suction. It can leave temporary bruised painful marks on the skin. There is a small risk of skin burns. In traditional Chinese medicine (TCM), cupping is a method of applying acupressure by creating a vacuum on the patient's skin to dispel stagnation (of blood and lymph), thereby improving energy (qi) flow. Practitioners of cupping believe this maneuver invigorates blood flow and promotes healing. Cupping is used on back, neck, shoulder, and other musculoskeletal conditions (Figure 2). Cupping is also claimed by proponents to treat a broad range of medical conditions such as blood disorders (anaemia, haemophilia), rheumatic diseases (arthritic joint and muscular conditions), fertility and gynaecological disorders, and skin problems (eczema, acne) and help general physical and psychological wellbeing [7–9]. 

In order to address the clinical question if cupping is useful for childhood eczema, a clinical search on PubMed using key words of “cupping,” “eczema,” and “atopic dermatitis” was performed. There was no limit set for age, gender, or search period. Only two relevant papers are identified [8, 10]. Yao and Li reported treatment of eighty-eight cases of acute eczema [10]. The treatment group (*n* = 46) was treated with pricking the affected parts with three-edge needle and blood-letting puncturing and cupping at back-shu points with three-edge needle and the control group (*n* = 42) with oral administration of Claritin and external application of Pairuisong Ointment. There was no difference in the “total effective rate” (presumably meaning any effect) but significant difference in the “cured and markedly effective rate” between the two groups. The authors conclude that pricking and blood-letting and cupping with a three-edge needle has a definite therapeutic effect on acute eczema, which is better than that of the Western medicine. The article was reported in Chinese. Chen and Yu performed an overview on acupuncture, electrostimulation, and reflex therapy in dermatology and commented that the main drawback for these treatment modalities was a lack of controlled studies [8]. There was inadequate original scientific data cited in the overview for reference. Conclusively, therapeutic efficacy was claimed but not scientifically documented in these reports.

Despite the very high IgE in this girl, there were no unusual facial features, recurrent candidal infections, or pneumonias to suggest hyper-IgE or Job syndrome (a heterogeneous group of immune disorders). Abnormal neutrophil chemotaxis due to decreased production of interferon gamma by T lymphocytes is thought to be associated with Job syndrome [11]. Both autosomal dominant and recessive inheritance have been described [12]. Job syndrome is linked to mutations in the STAT3 or DOCK8 pathways. Regardless if this girl has any of these genetic mutations, the principle of treatment for her eczema is the same. This case report agrees with published experience that cupping as a form of alternative therapy is not without harm [9]. Unfortunately, desperate caregivers of children with recalcitrant chronic skin disease such as eczema seek these risky and painful therapies with unproven value. The practice of treating eczema with unfamiliar topical herbal remedies should also be discouraged [13]. Cupping is particularly not advised over excoriated, oozing, or infected areas as many eczema patients are colonized with *S. aureus*.

Childhood eczema is an eminently treatable atopic disease. Extreme alternative therapy seems not to be efficacious and may even be associated with serious undesirable sequelae [14]. Physicians should also be aware of various alternative treatment modalities and be prepared to offer evidence-based advice to the patients with eczema and their families [9].
